# Total Talar Prosthesis, Learning from Experience, Two Reports of Total Talar Prosthesis after Talar Extrusion and Literature Review

**DOI:** 10.3390/medicina59081498

**Published:** 2023-08-21

**Authors:** Danilo Leonetti, Giorgio Carmelo Basile, Gabriele Giuca, Elena Corso, Domenico Fenga, Ilaria Sanzarello

**Affiliations:** 1 Department of Biomedical, Dental and Morphological and Functional Images, University of Messina, 98122 Messina, Italy; d.leonetti@unime.it (D.L.); dfenga@unime.it (D.F.); i.sanzarello@unime.it (I.S.); 2 Department of Human Pathology of Adult and Developmental Age “Gaetano Barresi”, Faculty of Medicine and Surgery, University of Messina, 98122 Messina, Italy; gabgiuca@unime.it (G.G.); elenacorso14@gmail.com (E.C.)

**Keywords:** custom made prosthesis, talar avascular necrosis, talar enucleation, talar extrusion, total talar prosthesis

## Abstract

Recently, total talar prosthesis has been proposed to substitute the talus during the management of complex talar lesions such as talar extrusion, comminuted talar fractures, or avascular necrosis. Herein, we report two cases of talar extrusion treated with total talar replacement after a high-intensity trauma. Both cases subsequently required revision surgery due to degenerative changes of the tibial plafond (arthrodesis in the first case, conversion to a total ankle prosthesis in the latter). We report and analyze the literature concerning total talar replacement to discuss strategies that could help improve prosthesis survival and reduce the incidence of osteoarthritis.

## 1. Introduction

In the last three decades, technical and technological advances have allowed surgeons to benefit from a variety of new options to manage complex lesions. Advances such as 3D printing allowed for the re-creation of anatomical structures with custom-made prostheses [1]. In recent years, total talar replacement (TTR) has been proposed as a treatment for complex talar injuries. Talar lesions (such as high-grade avascular necrosis, comminuted fractures, and severe osteoarthritis) represent a challenge for the orthopedic surgeon. Current management options are often burdened by unsatisfactory functional outcomes: Ankle arthrodesis, the most adopted, guarantees pain reduction and stability but can nevertheless lead to dysmetria and secondary osteoarthritis at the adjacent joints. External fixation methods are also associated with a high rate of nonunion and infection, while internal fixation and minimally invasive surgeries account for these complications [2,3].

In 2007, Stevens et al. introduced the so-called third-generation talar prosthesis, reproducing anatomically the talus in its entirety, namely, the TTR [4]. TTR can be isolated or, if the talar prosthesis articulates with a distal tibial component, combined. Combined TTR is conceptually a total ankle replacement with a total talar prosthesis.

Talar extrusion—also known as talar enucleation/missing talus—stands out as one of the rarest and more complex talar injuries. It consists of the complete dislocation of the talus from the tibiotalar, talocalcaneal, and talonavicular joints [5], usually following a forced tibiotalar plantar flexion combined with excessive supination. It is determined by high-energy trauma and has been described 91 times in the literature [6,7,8,9,10,11,12,13,14]. Due to their peculiar and limited blood supply, talar dislocations are difficult to treat: Re-implantation of the extruded talus is possible but frequently presents fearsome complications such as avascular necrosis, infections, and osteoarthritis [7,8,15]. Historically, primary talectomy or (in selected patients) tibio-calcaneal arthrodesis with the Blair fusion technique was taken into account, although it was burdened by a high rate of complications and reduced functional outcomes, such as loss of function of the peri-talar joints, shortening of the injured leg, and the frequent insurgence of secondary degenerative changes [7,15,16].

As follows, we present, respectively, two cases of talar extrusion treated with custom-made total talar replacement (TTR). Both cases required revision surgery at the 2-year follow-up due to degenerative changes of the tibial plafond (arthrodesis in the first case, conversion to a total ankle prosthesis in the latter). We reviewed the literature regarding TTR. We discussed potential indications of TTR, benefits and drawbacks, and the most common causes of implant failure and surgical revision.

## 2. Cases Presentation

### 2.1. Case 1

#### 2.1.1. Talar Extrusion

**Figure 1:**
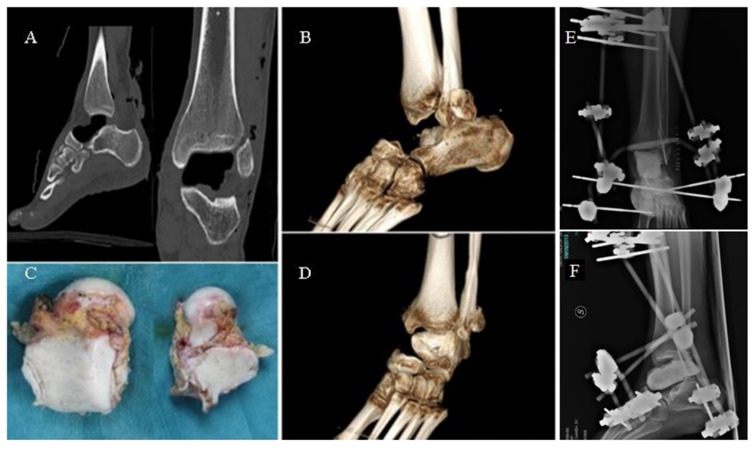
CT images (**A**) and volume rendering (**B**,**D**) showing the gap left by the talus (**C**). External fixation of the ankle joint (**E**,**F**).

A 27-year-old male patient suffered a road accident in September 2013, reporting a superficial traumatic head injury and a left fibular fracture with an open wound and complete enucleation of the left talus. The missing talus was collected: It presented minor osteochondral injuries in the medial part and macroscopic contamination but was not fractured. After primary care, the wound on the foot and ankle was washed and debrided, and an antibiotic-coated cement spacer was applied to fill the void left by the talar enucleation (Figure 1). The fibula was stabilized using K-wires, and an external fixator was applied to maintain the stability of the ankle. The talar void was filled by a gentamicin/clindamycin-loaded cement spacer (Figure 1).

#### 2.1.2. Failure of Talar Reimplantation

**Figure 2:**
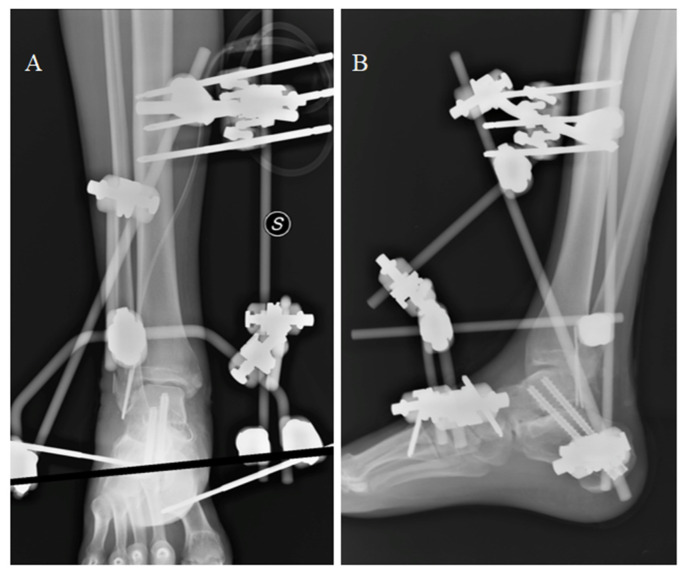
Talar reimplantation with subtalar arthrodesis: anteroposterior (**A**) and latero-lateral (**B**) X-ray.

The extruded talus was sent to the Musculoskeletal Tissue Bank of the Rizzoli Institute (Bologna), where it underwent cleaning and sterilization by gamma irradiation. After 21 days, cement was removed and the original talus was implanted with an anterior approach and a subtalar arthrodesis (Figure 2), as described by Vaienti et al. [15].

**Figure 3:**
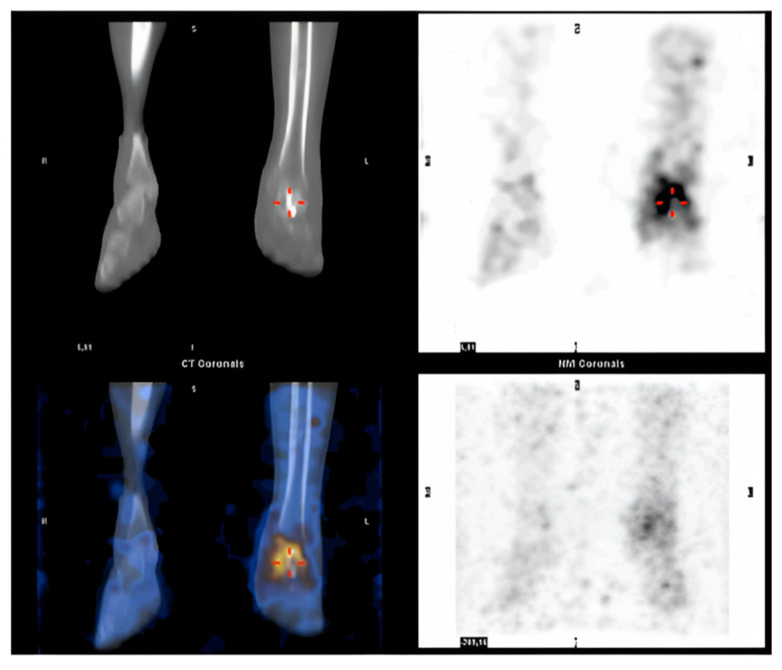
SPECT CT suggesting a deep tissue infection of the reimplanted talus. Red hyphens point out the site of infection.

Post-operative recovery was uneventful. After two months, the patient started to complain of ankle pain and swelling: Single-photon emission computed tomography (SPECT-CT) confirmed the clinical suspicion of a deep tissue infection involving the reimplanted talus (Figure 3). The talus was removed and replaced by a new spacer in antibiotic-coated cement. A negative pressure treatment was applied to facilitate soft tissue healing.

#### 2.1.3. Total Talar Replacement

**Figure 4:**
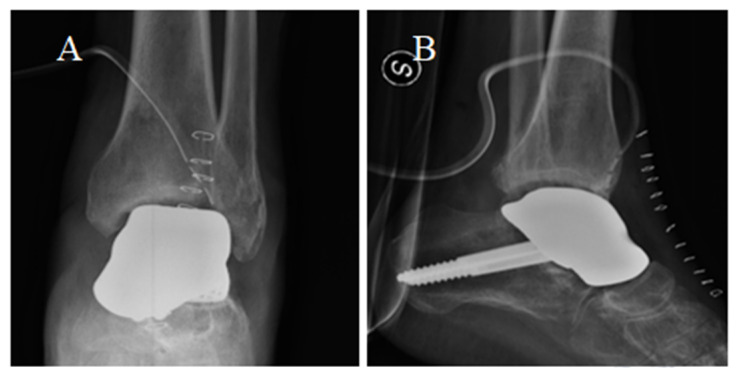
Total talar replacement with subtalar fixation, post-operatory anteroposterior (**A**) and latero-lateral (**B**) X-ray.

After the failure of re-implantation, two therapeutic options (arthrodesis and TTR) were presented to the patient. The patient agreed to undergo the implantation of a talar prosthesis. The prosthesis was made by 3D printing the mirrored CT scan of the contralateral talus. The customized implant was produced by casting a chromium-cobalt alloy (Sintac Srl, Trento, Italy). The talar prosthesis was manufactured using state-of-the-art laser technology by powder melting a cobalt-chromium alloy (nickel, beryllium, and cadmium free, according to standard DIN-EN-ISO 22674:2006) and included porous articular surfaces and a talar-navicular component with two channels to host the lag screws for subtalar fixation. The implant weight was 390 g. In May 2014, after complete healing of the soft tissues, the customized talar prosthesis was implanted: After an anterior-medial approach, the spacer in antibiotic-coated cement was exposed and removed. A thorough soft tissue debridement was performed, and the articular cartilage was removed from the subtalar calcaneal surface. Subsequently, the tailor-made talar prosthesis was implanted and fixed to the calcaneus by two screws (Figure 4).

**Figure 5:**
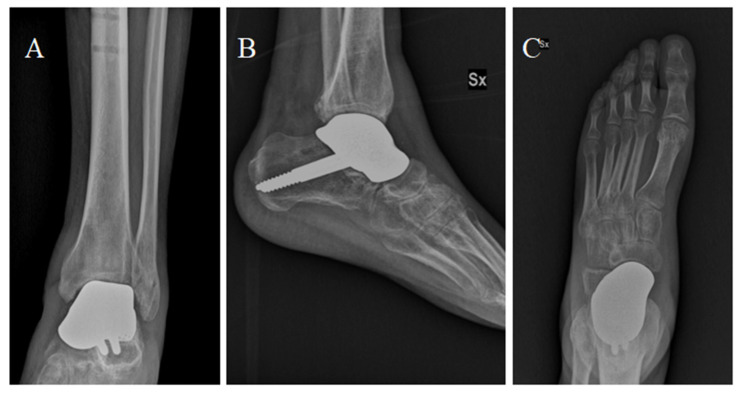
Anterior-posterior (**A**), dorsoplantar (**B**) and latero-lateral (**C**) projections showing the implant at the 6-month follow-up after surgery.

Percutaneous Achilles tendon lengthening was performed to improve ankle dorsiflexion. After surgery, a plaster cast was applied for 3 weeks. After 3 weeks, partial weight bearing was allowed, with full weight bearing within 9 weeks of surgery. We decided to examine the patient at 1, 2, 4, 6, and 12 months after surgery, and then once a year (Figure 5). The patient was satisfied with the outcome of the surgery and showed good functional results: At 6 months, he presented an AOFAS (American Orthopedic Foot and Ankle Society Score) of 86 and an NRS (Numeric Rating Scale) of 2. The sagittal range of motion (ROM) was >30°. The patient satisfaction as well as the clinical scores were retained at the 12-month follow-up.

#### 2.1.4. Tibial Osteoarthritis and Arthrodesis

The patient started to complain of pain and functional limitations 24 months after surgery. At the physical exam, the patient presented a limp, and passive ROM on the sagittal plane was reduced to 20°. Ankle X-ray in latero-lateral view revealed osteoarthritic changes with osteophytes and sclerosis of the tibial subchondral bone.

**Figure 6:**
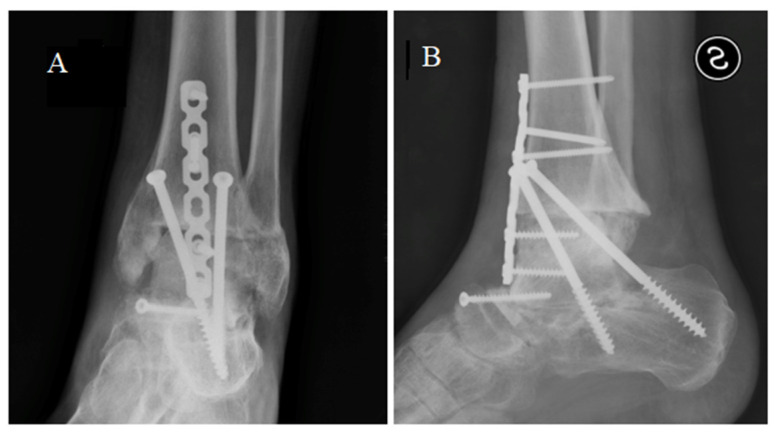
X-ray in anterior posterior (**A**) and latero-lateral (**B**) projections showing the tibio-calcaneal arthrodesis.

We proposed a tibial resurfacing, but the patient preferred a triple arthrodesis. Via anterior-medial access, the prosthesis was removed, and a fusion of the ankle, subtalar, and talo-navicular joint was performed with the use of a bone graft (Figure 6). At the last follow-up, 5 years after surgery, the patient was pain-free, wore normal shoes, and walked with a very slight limp. The AOFAS score was 81.

### 2.2. Case 2

#### 2.2.1. Talar Extrusion

**Figure 7:**
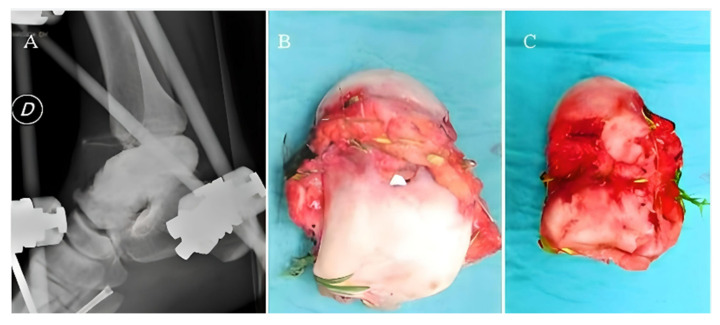
Latero-lateral ankle radiograph (**A**) shows the placement of the cement spacer and the ankle stabilized by external fixation. The talus as it appeared right after being collected from the site of the plane crush: a severe macroscopic contamination can be observed (**B**,**C**).

In June 2015, a 32-year-old male pilot was the victim of a plane crash, suffering from L2, L3, and L4 amyelic vertebral fractures, a fracture of the right ulna, a comminuted tibial plateau fracture of the right knee, a fracture of the 5th metatarsal bone of the right foot, and a Gustilo Anderson III C open fracture of the right ankle with complete enucleation of the talus, severe capsule-ligamentous lesion, and a tear of the extensor digitorum longus tendon and tibialis anterior artery. The patient underwent primary care, diagnostic investigations, and primary surgery. Approximately 6 h after the admission to the emergency room, the patient underwent surgical lavage and debridement of the ankle injury site with the application of an antibiotic-coated cement spacer. The ankle was stabilized by an external fixator. Meanwhile, the extruded talus was collected and sent to the Musculoskeletal Tissue Bank of the Rizzoli Institute (Bologna) for decontamination. Microbiological analysis revealed severe contamination by filamentous fungi and bacteria; thus, the infective risk for reimplantation was deemed too high (Figure 7).

#### 2.2.2. Total Talar Replacement

A TTR was planned and realized by the Canary Islands Institute of Technology as an exact reproduction of the shape and size of the original talus. A CT scan and volume rendering of the enucleated talus was sent to the institute, where the artificial talus was created by electron beam melting (EBM ARCAM S12, Arcam AB, Mölndal, Sweden). Electron beam melting is a 3D printing process effective and validated for the production of titanium orthopedic implants) [17].

The prosthetic implant was made of trabecular titanium Ti6Al4V, weighing only 78 g, with smooth and chrome-covered tibial, fibular, and navicular joint surfaces. The calcaneal surface was kept porous to ensure the best adhesion of the talo-calcaneal surfaces.

**Figure 8:**
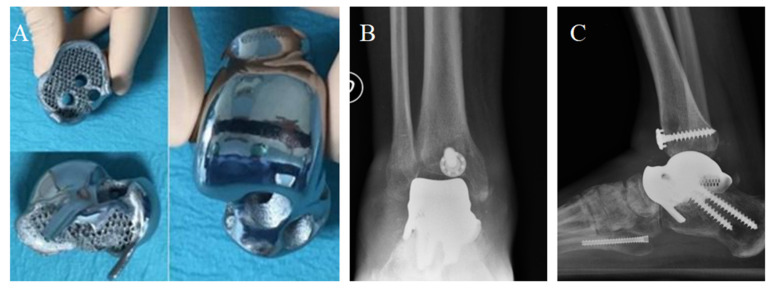
The prosthesis ready to be implanted (**A**). X-ray of the implant from antero-posterior (**B**) and latero-lateral (**C**) projections. Articular space can be observed in figure (**B**).

Four channels were carved at the level of the talus neck to fit the screws and allow ligament reconstruction. Two months after the accident, the prosthetic replacement and ligament reconstruction was finally performed by exposing the cement spacer through an anteromedial longitudinal approach. After cement removal, a customized guide was used to prepare the upper surface of the calcaneal bone according to preoperative planning. The talar prosthesis was fixed to the calcaneus by two screws. Antero-medial capsule-ligamentous reconstruction was performed using a peroneus brevis tendon allograft inserted at the level of the talus foramen and stabilized at the tibial level with a transosseous tunnel and Soft Tissue Anchoring System (CONMED) (Figure 8).

After surgery, the ankle was immobilized in a plaster cast for 3 weeks. At 6 weeks, the patient started gait re-education with progressive weight bearing and a bivalve brace. In 12 weeks, the patient started walking without any limitations. At 1 year follow-up, good radiographic results and a fair functional outcome were reported (AOFAS = 74; NRS = 2); total ROM was 30° with 10° of dorsiflexion and 20° of plantar flexion.

#### 2.2.3. Secondary Osteoarthritis and Revision to Total Ankle Prosthesis

**Figure 9:**
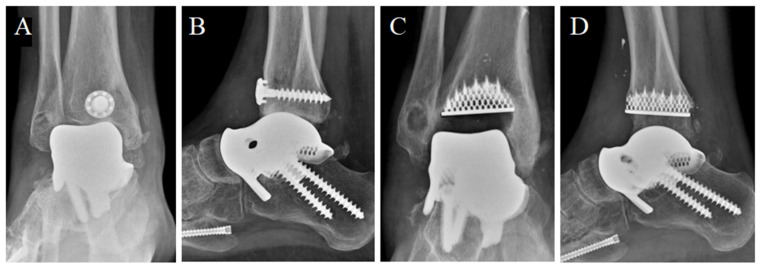
Two-year post-surgery X-ray of the implant from antero-posterior (**A**) and latero-lateral (**B**) projections. Articular space narrowing and osteophyte formation (compared with Figure 7) can be observed (**A**,**B**). The total talar replacement after revision surgery from antero-posterior (**C**) and latero-lateral (**D**) projections. *Image courtesy of Dr. Paola Verde Aerospace Medicine Department, Aerospace Test Division, Pratica di Mare, Rome, Italy.*

During the second year of follow-up, the patient experienced a worsening, up to the impossibility of walking without pain. He soon started complaining of painful plantar flexion and morning stiffness. A ROM limitation (ROM = 20°) was observed at the control visit. Two years after surgery, antero-posterior and latero-lateral ankle radiographs suggested a secondary osteoarthritis with articular space narrowing, implying the indication for a prosthesis revision (Figure 9). Either arthrodesis or tibial resurfacing were proposed as management options. In this case, the patient expressed the desire to maintain ankle function: A conversion to total ankle arthroplasty with TTR was performed. The tibial prosthesis consisted of a tibial trabecular titanium (Ti6Al4V) component and 6 mm-thick, high-density polyethylene (Figure 9). The lengthening of the Achilles tendon was performed using a percutaneous technique.

After surgery, the patient was enrolled in a rehabilitation program based on continuous passive movement; progressively, partial weight bearing was allowed. Total weight bearing was conceded after 3 months.

Conversion to total ankle arthroplasty achieved a positive outcome: After every 1-year follow-up (up to 5 years), the patient complained of little to no pain and reached 35–40° of total sagittal ROM. After one year, the patient was judged fit to fly, and after two years, he resumed flying on high-performance jets. The outcomes were stationary at the last follow-up, 5 years after surgery. The aforementioned revision surgery and the implications and observations concerning specifically Aerospace and Aviation Medicine have been presented and discussed in detail in the Journal of Aerospace Medicine and Human Performance by Verde and colleagues [18].

## 3. Materials and Methods

We conducted a literature review to summarize the current knowledge and scientific evidence regarding third-generation TTR. We conducted our search in the following databases from the beginning until November 2021: PubMed, Google Scholar, and MEDLINE. The search strategy was developed and executed in the mentioned research databases with the following queries:

1. (talus [MeSH Terms]) AND (prosthesis [MeSH Terms])
2. ((total talus) AND (total talar)) AND ((replacement) OR (prosthesis))

Following identification of potential articles, an initial screening of titles and abstracts that addressed the research question of interest was performed before inclusion.

## 4. Results

The query “(talus [MeSH Terms]) AND (prosthesis [MeSH Terms])” produced 464 results, while “((total talus) AND (total talar)) AND ((replacement) OR (prosthesis))” produced 154 results.

After a thorough literature review, we selected a total of 15 case reports (18 patients), 10 case series (119 patients), and 2 case series (64 patients), for a total of 201 cases.

In the studies analyzed, the average follow-up was 36.06 months (4.7–132 months, SD ± 16.16). Follow-up was reported in a heterogeneous manner, with several studies not reporting the follow-up time for every single patient but only as an average value.

Outcomes were measured by evaluation of post-operative total ROM, ROM in dorsiflexion, and ROM in plantar flexion: Often, studies did not specify if the evaluation was limited to active ROM or included both passive and active ROM. ROM was reported in 3 case reports and in 5 case series, with an average total ROM of 43.1°: an average dorsiflexion ROM of 11.7°, and an average plantar-flexion ROM of 34.0°.

To evaluate functional outcomes, a wide variety of scoring systems were adopted: The only two validated outcome measures adopted in >2 studies at the last follow-up were AOFAS (81.94 points; 23 patients) [10,19,20,21,22,23,24,25,26] and the Japanese Society for Surgery of the Foot scale (JSSF) (mean 89.68 points, 98 patients) [27,28,29,30,31]. Due to the diversity of the results obtained by the literature review, a statistical analysis was deemed not feasible. Results are therefore presented and discussed in narrative form.

## 5. Discussion

A customized talar prosthesis tends to reproduce as much as possible the tridimensional shape of the native talus. CT images of the original talus or 3D mirroring of the contralateral talus are often used as references. By reproducing the anatomic structure of the native talus, this prosthesis aims to overcome the main drawbacks of ankle arthrodesis (limb shortening, decreased shock absorption, and ROM limitation).

### 5.1. Case Reports

In the two cases discussed, the TTR showed satisfactory functional outcomes in the short term. Nevertheless, after approximately two years, the functional outcome was impaired by secondary arthritic degeneration of the tibial plafond, requiring secondary surgical revision. This is the first paper to report and discuss the failure of TTR and management strategies: In the first case, the patient preferred ankle arthrodesis, while in the second case, due to higher functional needs (high-performance aircraft pilot), a distal tibial resurfacing was performed. Ankle arthrodesis, the most adopted approach to treat complex talar injuries, guarantees pain reduction and stability of the ankle joint but often leads to unsatisfying functional outcomes, rigidity, dysmetria, and secondary osteoarthritis, commonly at the subtalar, talonavicular, calcaneocuboid, navicular-cuneiform, tarso metatarsal, and first metatarsophalangeal joints [2]. In the first case, the patient expressed the main desire to achieve ankle stability and pain reduction with a definitive solution, minimizing the risk for supplemental surgeries: ankle arthrodesis presents lower failure, complication, and reoperation rates when compared to total ankle arthroplasty [32].

The second patient needed optimal ankle functionality (with physiological ROM) to keep working as a military jet pilot. To avoid limb dysmetria and guarantee satisfactory sagittal ROM, there was a need to keep the talar prosthesis and create a tibial surface able to slide on the titanium without erosion. The material of the tibial prosthesis, Ti6A4V, exhibits tolerance to mechanical load and enhances maintenance and regulation of bone mass density due to its porous gyroid structure. This material guarantees good osteoconductive potential. A 6 mm-thick mobile ultra-high molecular weight polyethylene, such as in standard ankle replacements [33], was adopted with the aim of achieving approximately 40° of total sagittal ROM, necessary to resume flight activity. To prevent retraction after Achilles tendon lengthening, the continued passive motion of the operated ankle was programmed up to two months after surgery.

### 5.2. Literature Revision

#### 5.2.1. Main Indications

In managing patients with severe talar lesions, loss of the original bone, or poor bone stock, such as avascular necrosis, advanced osteoarthritis, rheumatoid arthritis, or bone tumors, TTR could be part of a total ankle replacement [27,28,29]. In 2020, Morita and colleagues presented a case series of 10 total ankle arthroplasties revised for subtalar subsidence with the implant of a TTR. All patients significantly improved their ROM and NRS scores and returned to activities of daily living at a long-term follow-up [29]. The authors suggested that TTR could be an option to address unfavorable but common complications of total ankle replacements, such as talar component subsidence, in selected patients with large bone defects. Studies comparing the long-term outcomes of total ankle replacement with those of combined TTR are still missing.

TTR has been used to treat severe ankle injuries with loss or irreversible damage to the talar bone. In severe comminuted fractures, where simple open or closed reduction is burdened by the risk of aseptic necrosis, arthritis, and pseudarthrosis postoperatively [19]. TTR guarantees good congruency with adjacent joints and preserves leg length and ankle mobility. In a 2015 case report, Giannini and colleagues provided a remarkable example of TTR adopted to achieve valid functional outcomes in a 27-year-old rock climber with high functional requirements and poor bone quality: The patient was suffering severe osteoarthritis and talar osteonecrosis following an open reduction and internal fixation of a talonavicular fracture. The implant of a TTR (including the navicular bone) achieved optimal results: He resumed alpine skiing, climbing, running, and even became a climbing instructor. In this case, specifically, these functional results were otherwise impossible to achieve: insufficient bone support for implant integration contraindicated a typical total ankle arthroplasty [22].

Katsui and colleagues described a series of six severely comminuted talar fractures; again, TTR allowed to avoid arthrodesis: in this case, three patients resumed sports activities (golf, aerobics, and even jogging) [19].

Gadkari et al. and Stevens et al. reported the case of a 14-year-old female who suffered a talar extrusion and underwent a talectomy after a failed talar reimplantation. A TTR was the only option to avoid arthrodesis: In this case, the patient achieved a good ROM and was capable of walking on uneven terrain [4,21]. Finally, as previously reported, in the second of our cases, the combined implant was necessary to resume high-performance jet driving.

#### 5.2.2. Clinical Outcomes

As mentioned in the results, TTR demonstrates favorable functional outcomes. The mean AOFAS and JSSF scores at the last follow-up were comparable to or better than those achieved with ankle arthrodesis or total ankle replacement [34,35]. The attained range of motion (ROM) enables patients to perform daily activities and successfully resume sports participation in many cases [18,28,31].

#### 5.2.3. Cartilage Degeneration and Secondary Osteoarthritis

Like the two cases reported, the majority of TTR described worldwide are isolated. In 2015, Taniguchi and colleagues published a series of 55 isolated TTR with a minimum follow-up of 24 months (follow-up range: 24–96 months): 24 patients (44%) total presented tibial plafond osteoarthritis at the last follow-up. Additionally, 5 patients (9%) presented osteosclerosis of the navicular bone, and 19 patients (35%) had osteosclerosis of the calcaneus [36].

Among 42 combined TTR (with a follow-up >24 months), only 2 cases of degenerative changes were reported: specifically, in the work by Morita et al., 1 case of osteosclerosis at the talonavicular joint was reported, while 1 out of 3 patients described by West and colleagues presented loosening of the tibial component [29,37].

It is well known that the contact of native cartilage with prosthetic material could lead to degeneration and revision surgery. In total knee arthroplasty, direct metal contact on cartilage leads to long-term degeneration and suffering of the subchondral bone [38]. Several meta-analyses state that the risk of reoperation for hip hemiarthroplasty is higher than the one for total hip arthroplasty at long-term follow-up (over 24 months), often because of thigh pain and loss of function determined by acetabular cartilage degeneration [39,40,41]. Being the tibial plafond the most common site of osteoarthritis, we may speculate that choosing combined over isolated TTR (preventing tibial plafond osteosclerosis) could decrease the incidence of prosthesis revisions. In our second case (and in the one reported by Katsui et al.), a combined TTR could have eventually avoided a secondary surgery [19]. It is worth noting that the only study that compares total ankle arthroplasty with combined TTR showed that the latter achieves equivalent pain reduction, and significantly superior functional outcomes showed in Table 1 [27].

### 5.3. Limitations

The knowledge concerning TTR, particularly combined design, is still preliminary. A limited number of non-comparative, retrospective studies (namely, case series/reports) have been published, providing a scarce level of evidence with a high risk of publication bias. Furthermore, the current literature is characterized by heterogeneity of reporting regarding outcome measures and follow-up length; therefore, synthesizing results from different studies could be complex and, to some extent, misleading.

Further studies are needed to describe the potential advantages of TTR, for example, compared with TAA in patients with poor bone stock. Long-term outcomes of TTR are scarcely described, with current studies focusing on limited follow-up times that do not allow estimating the survival of these implants. The case series/report design exposes it to a high risk of publication bias compared to prospective studies.

Our manuscript should be considered a narrative overview of the state of the art concerning TTR, but a systematic and pooled approach will be needed in the future, considering the growing body of literature on this topic.

## 6. Conclusions

Due to the limited number of cases described, the approach towards severe damage and talar bone loss is diverse and, to some extent, empirical. To date, only case report/series have been published.

In our experience, custom-made TTR could find indication as a component of a total ankle replacement, in particular, if the talar bone is lacking in quality, such as in severe avascular necrosis or osteoarthritis, and could reduce the risk of talar component and subtalar subsidence. It represents a management option when the integrity of the talar bone is compromised, for example, in high-grade ankle injuries with severely comminuted talar fractures, extrusion (a minor risk of failure over reimplantation), or bone tumors. TTR maintains congruency with the adjacent joints to achieve ankle stability, a functional ROM, and prevent dysmetria.

Osteoarthritic changes in the adjacent joints (most frequently at the tibial plafond) represent one of the main drawbacks of TTR and the main cause of long-term (>2 years) failure of the implants.

This is the first article to present two cases of failed isolated TTR and to specifically discuss the reasons behind the need for secondary surgery and the pros and cons of the two main revision options (arthrodesis and tibial plafond resurfacing).

Comparing the data reported in several case reports and series in the literature, a combined TTR could prevent tibial degenerative changes, reducing the incidence of osteoarthritis and the need for secondary surgery.

In conclusion, although talar replacement is a growing and challenging topic in orthopedic research, standardized and reproducible research is still missing. Further research, with systematically collected data and a follow-up of at least 24 months, is needed to allow direct comparison with other surgical options and to provide the scientific basis for a cost–benefit analysis.

## Figures and Tables

**Table 1:** Mean American Orthopedic Foot and Ankle Society Score (AOFAS) and Japanese Society for Surgery of the Foot scale (JSSF) mean scores at the last follow-up as reported in the cited papers. N.R. = not reported.

Author and Year	Number of Cases	Follow-Up (Months)	AOFAS	JSSF
**Taniguchi et al., 2015,** [25]	54	52.8	N.R.	89.1
**Katsui et al., 2019,** [11]	6	46	78.8	N.R.
**Kanzaki et al., 2019,** [10]	22	34.9	N.R.	91.5
**Morita et al., 2020,** [16]	10	49	N.R.	88.5
**Tonogai et al., 2017,** [28]	2	18	N.R.	95
**Kurokawa et al., 2019,** [13]	10	58	N.R.	89
**Mu et al., 2021,** [18]	9	23.17	79.67	N.R.
**Gadkari et al., 2013,** [6]	1	48	97	N.R.
**Ando et al., 2015,** [1]	1	24	90	N.R.
**Giannini et al., 2015,** [7]	1	30	81	N.R.
**Ruatti et al., 2017,** [21]	2	24	77	N.R.
**Fang et al., 2018,** [5]	1	6	91	N.R.
**Chinzei et al., 2017,** [3]	1	48	88	N.R.
**Hussain et al., 2020,** [8]	1	12	94	N.R.
**Mean value (total patients)**			**81.94 (23)**	**89.68 (98)**

## Data Availability

Not applicable.

## References

[1] Tack P., Victor J., Gemmel P., Annemans L. (2016). 3D-printing techniques in a medical setting: A systematic literature review. Biomed. Eng. Online.

[2] Ling J.S., Smyth N.A., Fraser E.J., Hogan M.V., Seaworth C.M., Ross K.A., Kennedy J.G. (2015). Investigating the relationship between ankle arthrodesis and adjacent-joint arthritis in the hindfoot a systematic review a systematic review. J. Bone Jt. Surg..

[3] Biz C., Hoxhaj B., Aldegheri R., Iacobellis C. (2016). Minimally Invasive Surgery for Tibiotalocalcaneal Arthrodesis Using a Retrograde Intramedullary Nail: Preliminary Results of an Innovative Modified Technique. J. Foot Ankle Surg..

[4] Stevens B.W., Dolan C.M., Anderson J.G., Bukrey C.D. (2007). Custom talar prosthesis after open talar extrusion in a pediatric patient. Foot Ankle Int..

[5] Moussa M.K., Bou Raad R., Ghanem I., Mansour O. (2020). Complete Extrusion of Talar Body Associated with Ipsilateral Floating Knee. Cureus.

[6] Magnan B., Facci E., Bartolozzi P. (2004). Traumatic loss of the talus treated with a talar body prosthesis and total ankle arthroplasty: A case report. J. Bone Jt. Surg-Ser. A.

[7] Weston J.T., Liu X., Wandtke M.E., Liu J., Ebraheim N.E. (2015). A Systematic Review of Total Dislocation of the Talus. Orthop. Surg..

[8] Ortiz-Cruz J.R., Ojeda Boscana I.L. (2019). Talar extrusion, a very rare sequela of trauma: A case report. Am. J. Case Rep..

[9] Lalchandani G.R., Hung N.J., Janghala A., Terry M., Morshed S. (2022). Total Talar and Navicular Extrusions A Case Report. JBJS Case Connect..

[10] Ruatti S., Corbet C., Boudissa M., Kerschbaumer G., Milaire M., Merloz P., Tonetti J. (2017). Total Talar Prosthesis Replacement after Talar Extrusion. J. Foot Ankle Surg..

[11] Genena A., Abouelela A. (2020). A Case Report of an Open Pan-Talar Dislocation Case Presentation. Cureus.

[12] Stirling P., MacKenzie S.P., Maempel J.F., McCann C., Ray R., Clement N.D., White T.O., Keating J.F. (2019). Patient-reported functional outcomes and health-related quality of life following fractures of the talus. Ann. R. Coll. Surg. Engl..

[13] Almaeen B.N., Elmaghrby I.S., Alnour M.K., Alrefeidi T.A., Adas S.M.A. (2020). Complete Revascularization of Reimplanted Talus After Isolated Total Talar Extrusion: A Case Report. Cureus.

[14] Kwak J., Heo S., Jung G. (2017). Six-year survival of reimplanted talus after isolated total talar extrusion: A case report. J. Med. Case Rep..

[15] Vaienti L., Maggi F., Gazzola R., Lanzani E. (2011). Therapeutic management of complicated talar extrusion: Literature review and case report. J. Orthop. Traumatol..

[16] Joshi A.K., Joshi C., Singh S., Singh V. (2012). Traumatic loss of talus: A rare injury. Foot.

[17] Tamayo J.A., Riascos M., Vargas C.A., Baena L.M. (2021). Additive manufacturing of Ti6Al4V alloy via electron beam melting for the development of implants for the biomedical industry. Heliyon.

[18] Verde P., Guardigli S., Morgagni F., Roberts S., Monopoli D., Scala A. (2020). Total Ankle Replacement in a Military Jet Pilot. Aerosp. Med. Hum. Perform..

[19] Katsui R., Takakura Y., Taniguchi A., Tanaka Y. (2020). Ceramic Artificial Talus as the Initial Treatment for Comminuted Talar Fractures. Foot Ankle Int..

[20] Ando Y., Yasui T., Isawa K., Tanaka S., Tanaka Y., Takakura Y. (2016). Total Talar Replacement for Idiopathic Necrosis of the Talus: A Case Report. J. Foot Ankle Surg..

[21] Gadkari K.P., Anderson J.G., Bohay D.R., Maskill J.D., Padley M.A., Behrend L.A. (2013). An Eleven-Year Follow-up of a Custom Talar Prosthesis After Open Talar Extrusion in an Adolescent Patient. JBJS Case Connect..

[22] Giannini S., Cadossi M., Mazzotti A., Ramponi L., Belvedere C., Leardini A. (2016). Custom-Made Total Talonavicular Replacement in a Professional Rock Climber. J. Foot Ankle Surg..

[23] Fang G., Wang C., Piao Y., Zhang L. (2016). Chondro-osseous respiratory epithelial adenomatoid hamartoma of the nasal cavity. Pediatr. Int..

[24] Hussain R.M. (2021). Metallic 3D Printed Total Talus Replacement: A Case Study. J. Foot Ankle Surg..

[25] Mu M.D., Yang Q.D., Chen W., Tao X., Zhang C.K., Zhang X., Xie M.M., Tang K.L. (2021). Three dimension printing talar prostheses for total replacement in talar necrosis and collapse. Int. Orthop..

[26] Chinzei N., Kanzaki N., Matsushita T., Matsumoto T., Hayashi S., Hoshino Y., Hashimoto S., Takayama K., Araki D., Kuroda R. (2018). Total ankle arthroplasty with total talar prosthesis for talar osteonecrosis with ankle osteoarthritis: A case report. J. Orthop. Sci..

[27] Kurokawa H., Taniguchi A., Morita S., Takakura Y., Tanaka Y. (2019). Total ankle arthroplasty incorporating a total talar prosthesis a comparative study against the standard total ankle arthroplasty. Bone Jt. J..

[28] Kanzaki N., Chinzei N., Yamamoto T., Yamashita T., Ibaraki K., Kuroda R. (2019). Clinical Outcomes of Total Ankle Arthroplasty With Total Talar Prosthesis. Foot Ankle Int..

[29] Morita S., Taniguchi A., Miyamoto T., Kurokawa H., Tanaka Y. (2020). Application of a Customized Total Talar Prosthesis for Revision Total Ankle Arthroplasty. JBJS Open Access.

[30] Tonogai I., Hamada D., Yamasaki Y., Wada K., Takasago T., Tsutsui T., Goto T., Sairyo K. (2017). Custom-Made Alumina Ceramic Total Talar Prosthesis for Idiopathic Aseptic Necrosis of the Talus: Report of Two Cases. Case Rep. Orthop..

[31] Taniguchi A., Tanaka Y. (2019). An Alumina Ceramic Total Talar Prosthesis for Avascular Necrosis of the Talus. Foot Ankle Clin..

[32] Lawton C.D., Butler B.A., Ii R.G.D., Prescott A., Kadakia A.R. (2017). Total ankle arthroplasty versus ankle arthrodesis—A comparison of outcomes over the last decade. J. Orthop. Surg. Res..

[33] Daras-ballester A., Vicent-carsi V., Ramirez-fuentes C. (2022). Polyethylene Fractures in Mobile-Bearing Total Ankle Arthroplasty: Report of 2 Cases. Foot Ankle Orthop..

[34] Teramoto A., Nozaka K., Kamiya T., Kashiwagura T., Shoji H., Watanabe K., Shimada Y., Yamashita T. (2020). The Journal of Foot & Ankle Surgery Screw Internal Fixation and Ilizarov External Fixation: A Comparison of Outcomes in Ankle Arthrodesis. J. Foot Ankle Surg..

[35] Shih C., Chen S., Huang P. (2020). Clinical Outcomes of Total Ankle Arthroplasty Versus Ankle Arthrodesis for the Treatment of End-Stage Ankle Arthritis in the Last Decade: A Systematic Review and Meta-analysis. J. Foot Ankle Surg..

[36] Taniguchi A., Takakura Y., Tanaka Y., Kurokawa H. (2015). An Alumina Ceramic Total Talar Prosthesis for Osteonecrosis of the Talus. J. Bone Jt. Surg..

[37] West T.A., Rush S.M. (2021). Total Talus Replacement: Case Series and Literature Review. J. Foot Ankle Surg..

[38] Khatod M., Inacio M.C.S., Bini S. (2013). Short-Term Outcomes of Unresurfaced Patellas in Total Knee Arthroplasty. J. Knee Surg..

[39] The Health Investigators (2019). Total Hip Arthroplasty or Hemiarthroplasty for Hip Fracture. N. Engl. J. Med..

[40] Burgers P.T.P.W., Geene A.R. (2012). Van Total hip arthroplasty versus hemiarthroplasty for displaced femoral neck fractures in the healthy elderly: A meta-analysis and systematic review of randomized trials. Int. Orthop..

[41] Hopley C., Stengel D., Ekkernkamp A., Wich M. (2010). Primary total hip arthroplasty versus hemiarthroplasty for displaced intracapsular hip fractures in older patients: Systematic review. BMJ.

